# Draft Whole-Genome Sequence of a “*Candidatus* Liberibacter asiaticus” Strain from Yunnan, China

**DOI:** 10.1128/MRA.01413-18

**Published:** 2019-01-17

**Authors:** Yanling Chen, Tao Li, Zheng Zheng, Meirong Xu, Xiaoling Deng

**Affiliations:** a Guangdong Province Key Laboratory of Microbial Signals and Disease Control, College of Agriculture, South China Agricultural University, Guangzhou, Guangdong, China; University of Maryland School of Medicine

## Abstract

The draft genome sequence of “Candidatus Liberibacter asiaticus” strain YNJS7C, isolated from a navel orange tree in Yunnan Province, China, is presented here. The YNJS7C strain has a genome size of 1,258,986 bp, with a G+C content of 36.6%, 1,174 predicted open reading frames, and 53 RNA genes.

## ANNOUNCEMENT

The unculturable phloem-limited alphaproteobacterium “Candidatus Liberibacter asiaticus” is associated with citrus huanglongbing (HLB; also called yellow shoot disease), one of the most devastating diseases in citrus production worldwide (1 – 3). HLB was first reported in the Pearl River Delta area of Guangdong Province in China nearly a century ago (3). As of 2018, HLB has spread and been found in 11 of 19 citrus-producing provinces in southern China (4). In China, HLB can occur in both low-altitude provinces, such as Guangdong, and high-altitude provinces, such as Yunnan Province (5). Previous studies have revealed the population variation of “Ca. Liberibacter asiaticus” from different geographical locations (6, 7). Due to the inability to culture this bacterium, the characterization of “Ca. Liberibacter asiaticus” has relied heavily on genome analyses. The genome sequence of a strain of “Ca. Liberibacter asiaticus” isolated from a low-altitude region (Guangdong Province, China) was reported previously (8). To further compare the “Ca. Liberibacter asiaticus” strains from different geographic locations, here, we report a draft whole-genome sequence of “Ca. Liberibacter asiaticus” strain YNJS7C from Yunnan Province, a high-altitude province.

“Ca. Liberibacter asiaticus” strain YNJS7C was first identified in a navel orange (Citrus sinensis L. Osbeck) tree showing typical HLB symptoms in Jianshui City (23°38′N, 102°49′E) of Yunnan Province, China. Total plant DNA was extracted from infected citrus leaf midribs using the plant DNA extraction kit (Omega Bio-tek, Norcross, GA, USA). Library preparation was performed using the Illumina TruSeq version 2 paired-end library preparation kit (300-bp insert size), and sequencing was carried out on the Illumina HiSeq platform (San Diego, CA, USA). Quality control filtering and trimming were conducted using CLC Genomics Workbench 9.5 (Qiagen, Hilden, Germany). The HiSeq sequencing generated a total of 7.87 × 10^7^ reads with an average size of 150 bp. Using the whole-genome sequence of “Ca. Liberibacter asiaticus” strain A4 (GenBank accession number CP010804) (8) and those of three “Ca. Liberibacter asiaticus” prophages (SC1, SC2, and P-JXGC-3) (9, 10) as references, we identified a total of 110,266 reads using the Bowtie 2 software (version 2.3.4.2) with default settings (end-to-end read alignment mode) (11). *De novo* assembly was performed using CLC Genomics Workbench 9.5 with default settings. The assembly generated a total of three contigs ranging from 31,541 bp to 1,187,865 bp, with ∼15× coverage. The draft genome sequence of “Ca. Liberibacter asiaticus” strain YNJS7C comprises 1,258,986 bp, with a G+C content of 36.6%. Annotation was performed using the RAST server (http://rast.nmpdr.org/) (12). The strain YNJS7C genome was predicted to have 1,174 open reading frames (ORFs) and 53 RNA genes. Prophage identification by comparing sequences with those of three prophages (SC1, SC2, and P-JXGC-3) revealed YNJS7C to have SC2-like (type 2) and P-JXGC-3-like (type 3) prophages.

### Data availability.

This whole-genome shotgun project has been deposited at DDBJ/ENA/GenBank under the accession number QXDO00000000. The version described in this paper is version QXDO01000000. The sequence data have been deposited at the NCBI Sequence Read Archive (SRA) under BioProject number PRJNA488522.
