# Erratum for Chen et al., ‘Draft Whole-Genome Sequence of a “*Candidatus* Liberibacter asiaticus” Strain from Yunnan, China’

**DOI:** 10.1128/MRA.00497-19

**Published:** 2019-05-23

**Authors:** Yanling Chen, Tao Li, Zheng Zheng, Meirong Xu, Xiaoling Deng

**Affiliations:** a Guangdong Province Key Laboratory of Microbial Signals and Disease Control, College of Agriculture, South China Agricultural University, Guangzhou, Guangdong, China

## ERRATUM

Volume 8, no. 3, e01413-18, 2019, https://doi.org/10.1128/MRA.01413-18. Page 2, line 2: “SC2-like (type 2)” should read “SC1-like (type 1).”

